# Mesenchymal stem cells and mesenchymal stem cell-derived exosomes: a promising strategy for treating retinal degenerative diseases

**DOI:** 10.1186/s10020-025-01120-w

**Published:** 2025-02-21

**Authors:** Wenjing An, Wenliang Zhang, Jia Qi, Weihui Xu, Yushan Long, Huan Qin, Kai Yao

**Affiliations:** 1 Institute of Visual Neuroscience and Stem Cell Engineering, Wuhan University of Science and Technology, Wuhan, 430065 China; 2 College of Life Sciences and Health, Wuhan University of Science and Technology, Wuhan, 430065 China

**Keywords:** Mesenchymal stem cell, Exosome, Retinal degenerative diseases, Regenerative medicine, Neuroprotective

## Abstract

Mesenchymal stem cells (MSCs) have emerged as a promising therapeutic strategy in regenerative medicine, demonstrating significant potential for clinical applications. Evidence suggests that MSCs not only exhibit multipotent differentiation potential but also exert critical therapeutic effects in retinal degenerative diseases via robust paracrine mechanisms. MSCs protect retinal cells from degenerative damage by modulating inflammation, inhibiting apoptosis, alleviating oxidative stress, and suppressing cell death pathways. Furthermore, MSCs contribute to retinal structural and functional stability by facilitating vascular remodeling and donating mitochondria to retinal cells. Of particular interest, MSC-derived exosomes have gained widespread attention as a compelling cell-free therapy. Owing to their potent anti-inflammatory, anti-apoptotic, and vascular-stabilizing properties, exosomes show significant promise for the treatment of retinal degenerative diseases.

## Introduction

In recent years, stem cells have garnered significant attention due to their remarkable potential in regenerative medicine. Among the diverse stem cell types, mesenchymal stem cells (MSCs) have emerged as a focal point of research due to their multipotent differentiation capacity, robust paracrine signaling, low immunogenicity, and extensive tissue availability. Recent studies on MSCs have broadened into various fields, including the nervous and cardiovascular systems, positioning MSCs as a promising approach for treating degenerative diseases (Piscioneri et al. 2015; Badyra et al. 2020; Guo et al. 2020).

**Fig. 1:**
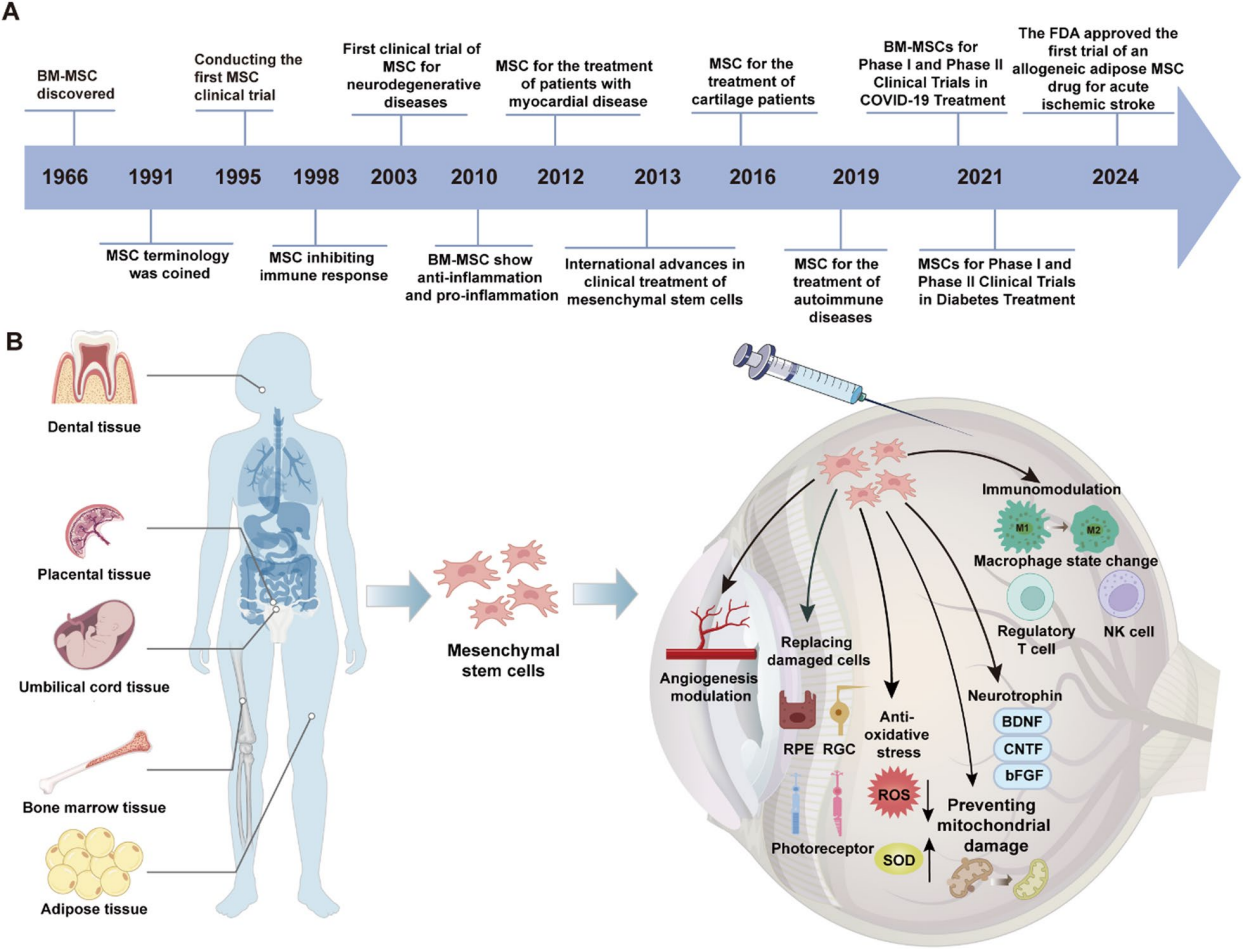
The research progress of MSCs and their role in the retina. **A** The historical timeline of mesenchymal stem cells (MSC) research and applications. **B** Schematic representation of MSC sources, including dental, placental, umbilical cord, bone marrow, and adipose tissues. MSCs exert therapeutic effects through immunomodulation, angiogenesis modulation, oxidative stress reduction, and secretion of neurotrophins (e.g., BDNF, CNTF, bFGF). They also enhance mitochondrial stability, promote tissue regeneration, and influence macrophage and regulatory T cell functions, contributing to their broad clinical potential

In 1970, Friedenstein and his colleagues first identified non-hematopoietic cells in mouse bone marrow capable of forming fibroblast-like colonies in vitro, initiating the field of MSC research (Fig. 1A). Subsequent research further refined the nomenclature and elucidated the biological properties of MSCs (Friedenstein et al. 1970; Phinney 2002). In 1999, a pivotal study first detailed the multipotent differentiation capacity of bone marrow-derived MSCs, which became a cornerstone for MSC identification and laid the scientific foundation for MSC-based therapies (Pittenger et al. 1999). Over the past few decades, MSCs have exhibited a wide array of biological functions, including multilineage differentiation, immunomodulation, angiogenesis, anti-apoptotic and anti-fibrotic activities, chemotaxis, and tissue repair, attracting considerable scientific interest (Konala et al. 2016). The International Society for Cellular Therapy (ISCT) defines MSCs as cells expressing CD105, CD73, and CD90, but lacking markers such as CD45, CD34, CD14, CD11b, CD79α, CD19, and HLA-DR (Dominici et al. 2006). Furthermore, MSCs possess the capacity for lineage differentiation into osteoblasts, adipocytes, and chondrocytes, while demonstrating plastic adherence characteristics in vitro (Mushahary et al. 2018; Abbaszadeh et al. 2022).

In recent years, MSC research has extended beyond bone marrow to encompass other tissues and organs, such as adipose tissue, umbilical cord, placenta, and dental pulp. MSCs derived from various tissues exhibit distinct biological properties, potentially addressing specific clinical requirements (Fig. 1B).

MSCs secrete substantial quantities of extracellular vesicles (EVs), which, functioning as paracrine mediators, confer benefits akin to MSCs and demonstrate comparable therapeutic efficacy in retinal diseases. These vesicles facilitate signal transduction with target tissues, modulate relevant pathways, and contribute to tissue repair (Anthony and Shiels 2013). MSC-derived exosomes facilitate the transition from cell-based therapies to acellular treatments. Due to their diminutive size, exosomes can efficiently traverse biological barriers, including the blood-retinal barrier, enabling targeted delivery to specific cells. Moreover, exosomes derived from MSCs are recognized as exceptional carriers for drugs or genes, positioning them as a valuable resource for both gene and cell-based therapies (Mead and Tomarev 2017). This also provides a more efficient and targeted approach to overcome the limitations inherent in traditional cell therapies for ocular diseases.

## Application of mesenchymal stem cells from different sources

Recent studies have shown that MSCs and their derivatives exhibit significant potential in the treatment of retinal diseases, emerging as potential therapeutic options for the treatment of diseases such as retinal degeneration, diabetic retinopathy and age-related macular degeneration (Pesaresi et al. 2021; Reboussin et al. 2022). The advantages of this therapeutic strategy mainly stem from the multiple biological properties possessed by MSC.

Bone marrow-derived mesenchymal stem cells (BM-MSCs) represent one of the most comprehensively investigated MSC subtypes. BM-MSCs originate from the stromal compartment of the bone marrow cavity and play a pivotal role in maintaining the bone marrow hematopoietic niche. Despite their limited abundance in bone marrow, BM-MSCs exhibit remarkable proliferative capacity, allowing for efficient large-scale expansion (Han et al. 2022). BMSCs hold substantial potential in regenerative medicine, particularly in modulating inflammation and enhancing skeletal repair, positioning them as key therapeutic agents for treating bone-related disorders through their ability to promote bone regeneration and mitigate inflammatory responses. (Zhang et al. 2012; Yuan et al. 2024). Within the realm of neural regeneration, BM-MSCs and their paracrine-mediated mechanisms have been shown to enhance neuroprotection and facilitate neural repair, offering a promising strategy for spinal cord injury and neurodegenerative disorders. The immunomodulatory and angiogenesis-promoting effects of BM-MSCs can help repair retinal damage, which makes them promising for the treatment of diabetic retinopathy (Gu et al. 2018).

Adipose-derived mesenchymal stem cells (ADSCs) represent a prominent MSC subtype, typically derived from adipose tissue through minimally invasive isolation techniques ADSCs exhibit robust differentiation capacity and remarkable paracrine activity, secreting a broad spectrum of growth factors, cytokines, and anti-inflammatory molecules. Evidence from in vitro studies and animal models highlights the substantial efficacy of ADSCs in tissue regeneration and immunomodulation (Wang et al. 2023; Gu et al. 2024). Due to their abundant availability, minimally invasive harvest methods, and superior proliferative capacity, ADSCs have gained significant traction in regenerative medicine, particularly in skin wound repair, anti-aging interventions, and angiogenesis research (Suh et al. 2019). ADSCs has strong proliferation ability and differentiation potential, and can effectively repair retinal injury (Rajashekhar 2014).

Umbilical cord-derived mesenchymal stem cells (UC-MSCs) have emerged as a focal point in regenerative medicine and immunotherapy, predominantly isolated from Wharton’s jelly and umbilical cord blood. Compared to bone marrow-derived MSCs, UC-MSCs provide distinct advantages, including non-invasive collection methods, enhanced safety profiles, and fewer ethical constraints. Evidence suggests that UC-MSCs possess potent immunomodulatory properties, including T cell suppression and regulation of inflammatory mediators, positioning them as promising therapeutic candidates for inflammatory, fibrotic, and immune-mediated disorders (Jiang et al. 2024; Yang et al. 2024b). Furthermore, UC-MSCs have gained considerable attention for their potential in treating neurological disorders, particularly brain and spinal cord injuries, by facilitating neuroregeneration and enhancing functional recovery via paracrine signaling (Zhai et al. 2022; Mu et al. 2024). They are able to improve retinal blood supply and promote neuroprotection and regeneration by secreting cytokines and growth factors (Zhang et al. 2017).

Placenta-derived mesenchymal stem cells (P-MSCs) are another important source of MSCs, predominantly derived from the chorionic and amniotic membranes of the placenta. Similar to umbilical cord-derived MSCs, P-MSCs demonstrate a robust proliferative capacity and exhibit potent immunomodulatory properties, with notable efficacy demonstrated in both in vitro and in vivo studies (Chen et al. 2023). P-MSCs have been extensively investigated for their therapeutic potential in treating inflammatory diseases, promoting tissue repair, and mitigating fibrosis. Furthermore, P-MSCs hold significant promise in anti-aging interventions and skin regeneration, highlighting their broad therapeutic applicability (Matsuoka et al. 2024). Placental MSC has strong anti-inflammatory and immunomodulatory effects, which can reduce inflammation and promote tissue repair during the repair process after retinal injury (Koh et al. 2020).

Dental pulp stem cells (DPSCs), derived from the dental pulp tissues of permanent and deciduous teeth, represent a unique MSC subtype originating from the neural crest. The distinctive dental pulp microenvironment endows DPSCs with exceptional proliferative and differentiation potential, establishing them as a pivotal focus in tissue engineering and regenerative medicine. Compared to other MSC subtypes, DPSCs exhibit enhanced proliferative capacity and neural differentiation potential, positioning them as a prominent research focus in neural repair and regeneration. DPSCs possess remarkable neuroprotective properties, establishing their therapeutic potential for central nervous system injuries and neurodegenerative disorders (Xiao and Tsutsui 2013; Jang et al. 2018). Furthermore, DPSCs have demonstrated broad therapeutic potential in dentistry, particularly in dentin and periodontal regeneration, as evidenced by promising outcomes in multiple preclinical studies (Li et al. 2023b). DPSCs are capable of strong neural differentiation, can differentiate into retinal nerve-like cells, and show strong regenerative ability in retinal nerve injury repair (Mohebichamkhorami et al. 2023).

While MSCs hold immense promise in regenerative medicine, their inherent limitations, including suboptimal secretory activity and inadequate tissue specificity, continue to constrain their clinical utility. Engineered mesenchymal stem cells (eMSCs) have emerged as a transformative approach to address these challenges, leveraging genetic modification, secretome enhancement, and biomaterial integration to unlock expanded therapeutic potential. The eMSCs leverage diverse strategies, such as genetic modification, biomaterial integration, and secretome optimization, to enhance their functional properties, thereby demonstrating enhanced clinical applicability (Shams et al. 2023). Genetic engineering enables eMSCs to amplify the secretion of key therapeutic factors, such as VEGF (vascular endothelial growth factor) and BDNF (brain-derived neurotrophic factor), thereby boosting their pro-angiogenic and neuroprotective capacities (Hegde et al. 2024). The genetic modulation of chemokine expression equips eMSCs with enhanced tissue-targeting capabilities, facilitating their precise homing to injury sites and effective involvement in localized repair (Singh et al. 2021). Furthermore, integrating eMSCs with advanced biomaterials not only substantially enhances their in vivo viability but also augments their directed differentiation into target cell types (Soleimannejad et al. 2017, 2018).

## Mesenchymal stem cells for regenerative medicine

MSCs, compared to other adult multipotent stem cells, are characterized by their diverse sources, ease of extraction, and minimal ethical concerns. Unlike embryonic stem cells, MSCs exhibit a substantially lower risk of teratoma formation and other severe side effects, underscoring their superior safety profile in clinical applications (Zhou et al. 2021).

MSCs possess an exceptional capacity for self-renewal, maintaining their population via mitosis and facilitating tissue regeneration and repair through differentiation into various somatic cell lineages or the secretion of bioactive molecules. Furthermore, their inherently low immunogenicity significantly boosts their therapeutic potential. In allogeneic transplantation settings, MSCs evade detection and destruction by the host immune system, thereby suppressing immune and inflammatory responses effectively (Shi et al. 2018). This unique immunomodulatory property opens transformative opportunities for treating immune-related disorders.

Moreover, MSCs demonstrate remarkable therapeutic potential through their paracrine mechanisms, which involve the secretion of a highly complex and dynamic array of bioactive molecules. Their secretome includes cytokines, chemokines, and growth factors, alongside proteins, microRNAs, and peptides delivered via extracellular vesicles (Mabotuwana et al. 2022). These secreted factors are pivotal not only in enhancing cell survival but also in promoting tissue repair and functional recovery, thereby expanding the clinical utility of MSCs. Given these extraordinary attributes, MSCs are widely recognized as one of the most promising cell types in the field of regenerative medicine.

In recent years, MSC research has transitioned beyond traditional domains such as bone and cartilage repair, immunomodulation, and wound healing, delving into the exploration of intricate pathological mechanisms underlying neurological disorders (Andrzejewska et al. 2021; Xu et al. 2024a). The application of MSCs in the field of ophthalmology, particularly in corneal diseases, is undergoing extensive research (Ghiasi et al. 2023). Studies have shown that MSCs can differentiate into corneal epithelial cells, stromal cells and endothelial cells, thereby promoting corneal regeneration and repair (Ghiasi et al. 2021). These studies suggest that MSCs offer a potential treatment in the face of a shortage of corneal donors, and are expected to be an important treatment in the future to improve patients' vision and enhance their quality of life. Meanwhile, the therapeutic potential of MSCs for retinal degenerative diseases has drawn increasing attention (Adak et al. 2021). Retinal degenerative diseases are a principal cause of irreversible vision loss, with existing pharmacological and surgical approaches offering limited effectiveness. As an advanced therapeutic modality, cell-based therapy brings renewed hope to affected patients. MSCs exhibit a unique array of biological characteristics, including the capacity to transdifferentiate into neuron-like cells, self-renewal, promotion of cellular proliferation, anti-inflammatory and anti-apoptotic functions, and neuroprotective effects. These properties position MSCs as a highly promising candidate for the treatment of retinal degenerative diseases, providing innovative perspectives for cell-based therapy and emerging as a prominent research focus.

### Retinal degenerative disease

**Fig. 2:**
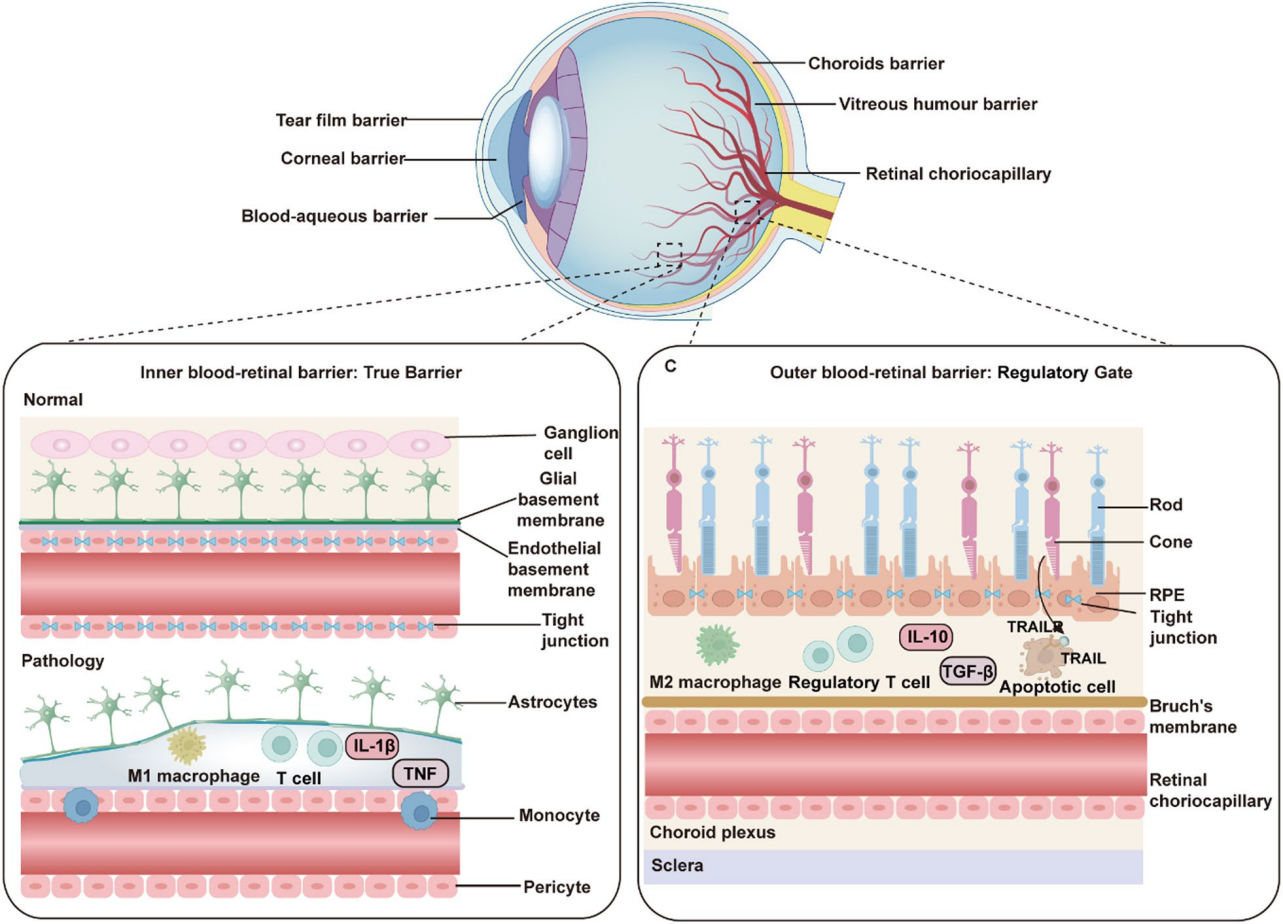
The functional components of ocular barriers. Ocular barriers include the conjunctival barrier, tear film barrier, corneal barrier, blood-aqueous barrier, vitreous humor barrier, and retinal choriocapillary barrier. The inner blood-retina barrier (BRB) acts as a true physical barrier, comprising tight junctions between endothelial cells, supported by glial cells and pericytes. The outer BRB functions as a regulatory gate, with the retinal pigment epithelial (RPE) forming tight junctions that control nutrient exchange

Vision serves as the dominant modality through which humans acquire environmental information, accounting for over 80% of sensory input (Liu et al. 2022a). However, there are approximately 43.3 million blind people and 295 million people with moderate or severe visual impairment worldwide, a large proportion of whom have partial vision loss due to retinal degenerative diseases (GBD 2019 Blindness and Vision Impairment Collaborators 2021). The blood-retina barrier (BRB), comprising tightly interconnected endothelial cells, a basement membrane, and glial end-feet, serves to restrict immune cells and macromolecules from entering the subretinal space, thereby preserving the immune-privileged microenvironment of the retina (Kashani et al. 2018) (Fig. 2).

**Fig. 3:**
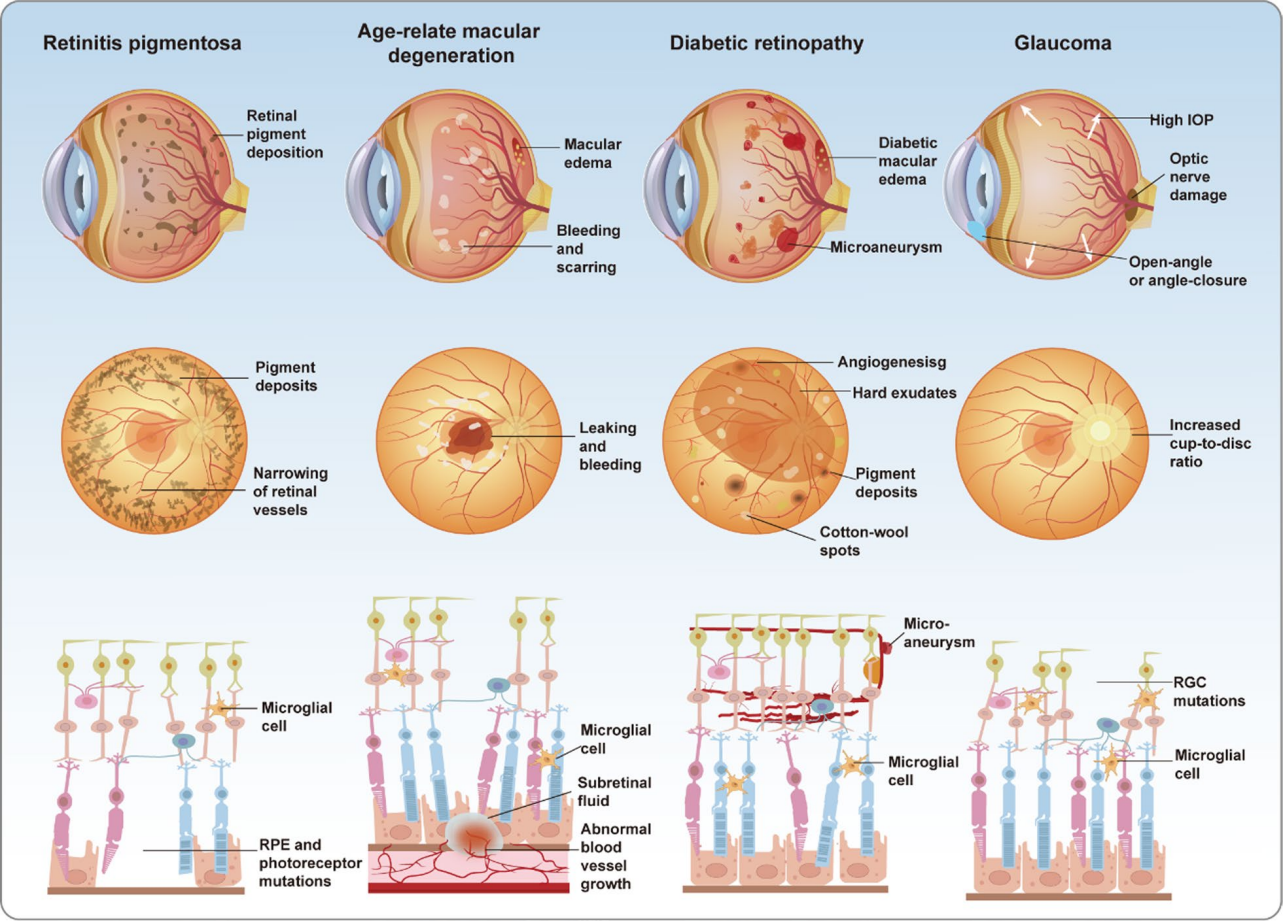
Pathological differences among four major retinal diseases. Retinitis pigmentosa (RP), progressive vision loss is caused by retinal pigment deposits, photoreceptor degeneration, and microglial activation. Age-related macular degeneration (AMD) is characterized by subretinal fluid, abnormal vascular growth, and macular damage, leading to central vision impairment. Diabetic retinopathy (DR) manifests as microaneurysms, vascular leakage, and neovascularization, accompanied AMD by inflammation and retinal edema. In glaucoma, elevated intraocular pressure (IOP) results in retinal ganglion cell apoptosis, optic nerve damage, and visual field loss

Retinal degenerative diseases, such as retinitis pigmentosa (RP), age-related macular degeneration (AMD), and diabetic retinopathy (DR), represent major causes of blindness and profound vision loss. Glaucoma, broadly defined, is likewise categorized as a retinal degenerative condition (Fig. 3). These disorders are often driven by a combination of aging, genetic predisposition, and environmental influences, culminating in the progressive dysfunction and loss of retinal cells. Such degeneration severely diminishes the eye’s capacity to perceive external stimuli and can ultimately lead to blindness (Berry et al. 2008). As a component of the central nervous system (CNS), the retina is irreversibly damaged upon neuronal death due to its inability to regenerate (Bertelli et al. 2020). The burden of vision loss resulting from retinal degenerative diseases is intensifying with the acceleration of industrialization and population aging.

Retinitis pigmentosa is a hereditary disorder defined by the progressive degeneration of rod and cone photoreceptor cells, representing a major cause of gradual vision loss among young and middle-aged populations. The hallmark pathological features of RP involve the disruption of photoreceptor outer segments, progressive thinning of the outer retinal layers, and functional impairment of retinal pigment epithelial (RPE) cells, often accompanied by occlusion of retinal microvasculature and the deposition of characteristic bone spicule-like pigments. The clinical severity of RP stems from its insidious onset and protracted progression, wherein patients typically manifest pronounced symptoms only at advanced stages, thereby restricting timely therapeutic intervention (Dias et al. 2018).

Age-related macular degeneration represents the foremost cause of irreversible vision loss in the elderly, primarily defined by the progressive degeneration of macular photoreceptors and RPE cells, culminating in marked central vision loss (Fleckenstein et al. 2024). Clinically, AMD is marked by central vision blurring and metamorphopsia, with advanced cases leading to total blindness and a profound impact on daily life. Pathologically, dry AMD manifests as RPE atrophy and drusen accumulation, whereas wet AMD is defined by aberrant choroidal neovascularization (CNV), often accompanied by retinal exudation, hemorrhage, and fibrotic scarring (Ramkumar et al. 2010). These pathological alterations are largely irreversible and advance rapidly, markedly elevating the risk of blindness among affected individuals.

Diabetic retinopathy represents a major cause of vision loss among working-age individuals, with an estimated one-third of diabetic patients experiencing retinal complications. Chronic hyperglycemia-induced abnormalities in retinal microvasculature form the central pathological basis of DR. Clinically, patients typically present with blurred vision, metamorphopsia, and, in advanced stages, sudden blindness (Vujosevic et al. 2020). The pathological hallmarks of DR encompass pericyte loss, microaneurysm formation, vascular leakage, and retinal edema. Disease progression is marked by abnormal neovascularization, which may cause vitreous hemorrhage, fibrosis, and tractional retinal detachment, culminating in blindness (Antonetti et al. 2021). The escalating global prevalence of diabetes has significantly amplified the public health burden of DR and its vision-threatening sequelae.

Glaucoma encompasses a spectrum of retinal neurodegenerative diseases defined by progressive optic neuropathy and the gradual constriction of visual fields, ranking among the leading causes of blindness globally. The multifaceted pathogenesis of glaucoma integrates abnormal intraocular pressure, mechanical deformation of the optic nerve head, axonal transport disruption, ischemia, oxidative stress, and inflammation (Alarcon-Martinez et al. 2023). These converging factors precipitate the progressive degeneration and loss of retinal ganglion cells (RGCs), culminating in irreversible optic neuropathy. The asymptomatic onset and insidious progression of glaucoma render it a formidable challenge to global vision health.

## Application of mesenchymal stem cells in the treatment of retinal degenerative diseases

**Fig. 4:**
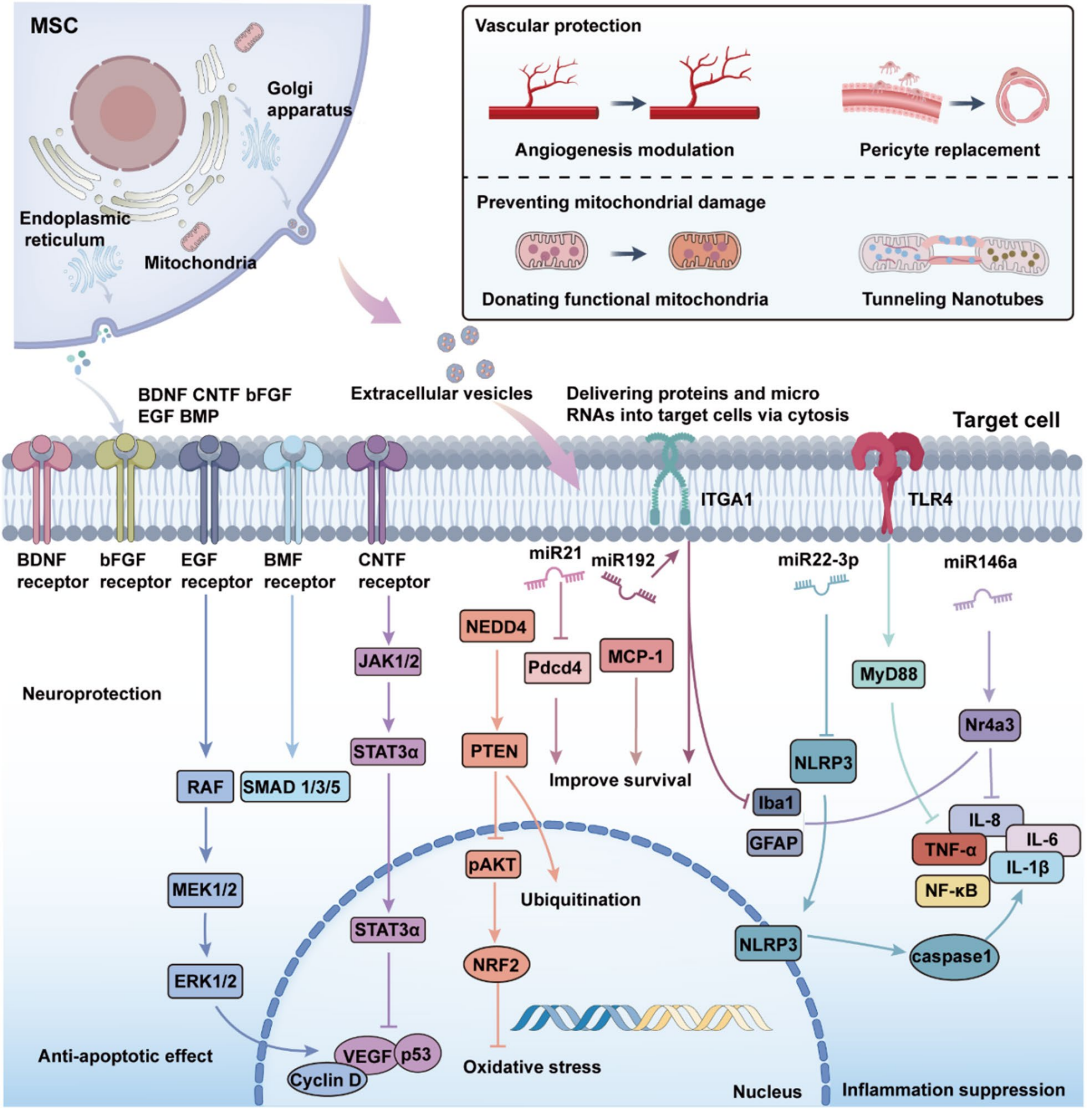
The mechanisms by which MSCs and their secreted factors contribute to retinal protection and regeneration. MSCs release neurotrophic factors and extracellular vesicles, delivering proteins and microRNAs into target cells to mediate therapeutic effects. Neuroprotection, achieved through receptor-mediated activation of JAK, STAT3, RAF, MEK, ERK, and SMAD pathways, promoting cell survival and reducing apoptosis. Vascular protection, involving angiogenesis modulation, pericyte replacement, and mitochondrial donation via TNTs; Anti-apoptotic effects, through PTEN, pAKT signaling and reduction of oxidative stress by NRF2 activation. Inflammation suppression, regulated by microRNAs (e.g., miR-21, miR-146a) and inhibition of pro-inflammatory pathways such as NF-κB, TNF-α, and NLRP3 inflammasome activation. These mechanisms collectively protect retinal cells from degeneration and promote tissue repair

Retinal degenerative diseases are associated with multifactorial mechanisms, including inflammation, oxidative stress, and apoptosis, which collectively exacerbate retinal injury and disrupt the retinal microenvironment. Advances in medical technology, particularly gene therapy and cell-based therapies, have opened new avenues for treating retinal degenerative diseases (Xu et al. 2024b). Both approaches are now in clinical trials, demonstrating their potential to repair retinal damage (Usategui-Martín et al. 2020). Gene therapy offers a precise approach to targeting inherited retinal disorders. Utilizing engineered adeno-associated virus (AAV) vectors, it enables effective expression of target genes, ensuring the production of functional proteins and the restoration of cellular function (Bucher et al. 2021). In cell-based therapies, stem cells exhibit notable advantages by fostering tissue regeneration (Liu et al. 2016). In recent years, the distinctive properties and extensive therapeutic potential of MSCs have garnered increasing attention. MSC-based cellular therapies have the potential to salvage the diseased retina through a number of different pathways, and therefore represent a promising new treatment for retinal degenerative diseases (Fig. 4).

### MSC differentiation replaces damaged retinal cells

Retinal degenerative diseases are frequently associated with the damage or loss of retinal neurons and RPE cells. Under normal physiological conditions, certain human retinal cell types, such as photoreceptors, are widely regarded as non-regenerative (Zuzic et al. 2022). However, under the influence of specific endogenous and exogenous factors, these cells may exhibit a limited capacity for regeneration during certain stages (Yao et al. 2018). In the study of treatments for retinal degenerative diseases, MSCs have become a major focus. Though the differentiation of MSCs into specific retinal cell types remains under investigation, accumulating experimental evidence indicates that MSCs can be induced to differentiate into retinal-relevant cells, including RPE cells, photoreceptors, and retinal ganglion cells, under defined conditions. This advancement offers a promising cellular source and paves the way for novel strategies in retinal repair for retinal degenerative diseases.

A study demonstrated that rat bone marrow-derived MSCs, transplanted into a sodium iodate-induced retinal injury model, differentiated into RPE cells, photoreceptors, and glial cells, highlighting their potential for retinal repair (Gong et al. 2008). Furthermore, researchers successfully induced human adipose tissue-derived MSCs to differentiate into RPE-like cells through the application of RPE-conditioned medium and gut peptide signaling molecules (Vossmerbaeumer et al. 2009). Studies further reveal that the inhibition of miR-410 markedly promotes the differentiation of umbilical cord blood-derived MSCs into RPE cells, with concurrent overexpression of RPE-specific markers. The differentiated cells display characteristic RPE morphology and functionality (Choi et al. 2015, 2017).

In additional experiments, *PAX6(5a)* gene transfection induced human adipose tissue-derived MSCs to rapidly acquire neuronal characteristics within 30 h, differentiating into retinal progenitor cells, RPE cells, and photoreceptors (Rezanejad et al. 2014). Moreover, the combined application of retinoic acid (RA) and taurine significantly upregulated neuroretinal and photoreceptor markers, facilitating the in vitro differentiation of MSCs into cone cells and retinal ganglion cells (Forouzanfar et al. 2021). MSCs originating from the trabecular meshwork exhibited the capacity to differentiate into functional photoreceptor-like cells when cultured on amniotic tissue (Nadri et al. 2013).

Multiple microRNAs, including MIR96 and miRNA-203, have been implicated in regulating the differentiation of MSCs into photoreceptor cells (Choi et al. 2016; Mahmoudian-Sani et al. 2019; Mahmoudian-Sani et al. 2024). Furthermore, GAP-43 has been identified as a key regulator in inducing bone marrow-derived MSCs to differentiate into retinal ganglion cells while activating neuroregenerative markers, underscoring the potential of MSCs in retinal repair (Wang et al. 2022). These findings collectively highlight that MSCs can differentiate into retinal cells under defined culture conditions, providing a promising strategy for cell replacement therapies in retinal degenerative disease treatment.

### Protective effect of paracrine of MSC on retina

Paracrine signaling has been identified as a key therapeutic mechanism of MSCs. MSC-derived secretome includes cytokines, chemokines, growth factors, and exosomes (Holan et al. 2021; Han et al. 2022). These paracrine factors enhance retinal cell survival, mitigate inflammation, and accelerate tissue repair through diverse molecular pathways. MSCs mitigate neurodegeneration in retinal diseases and facilitate cell survival and repair by releasing a broad spectrum of growth and neuroprotective factors, including BDNF, CNTF, bFGF, GDNF, HGF, and NGF (Zwart et al. 2009; Zhang and Wang 2010; Usategui-Martín et al. 2020; Fu et al. 2023). For example, the supernatant from light-damaged retinas markedly stimulates MSC-derived neurotrophic factor secretion, effectively attenuating apoptosis in damaged retinal cells (Lin and Xu 2018). MSCs also enhance endogenous CNTF expression via IL-6 signaling, contributing to neuroprotection (Heo et al. 2018). PDGF secreted by MSCs enhances retinal ganglion cell survival through the activation of intracellular neuroprotective signaling pathways, including AKT, ERK, and STAT3 (Osborne et al. 2018). The interplay of these mechanisms provides a synergistic foundation for developing novel therapeutic strategies targeting retinal degenerative diseases. Consequently, harnessing the paracrine mechanisms of MSCs offers substantial promise for the development of innovative cell-based therapeutic approaches.

### Anti-inflammatory and immunomodulatory effects of MSC

MSCs exert potent anti-inflammatory effects through diverse mechanisms. By secreting anti-inflammatory factors such as IL-10, IL-6, TGF-β, nitric oxide (NO), and hepatocyte growth factor (HGF), MSCs regulate immune responses, suppress the activation and proliferation of T cells and macrophages, and attenuate the release of pro-inflammatory cytokines, thereby protecting retinal cells from damage (Ghannam et al. 2010; Hermankova et al. 2019; Agrawal et al. 2021). Microglia, as the principal immune cells in the retina, become activated in response to retinal inflammation or injury. MSCs modulate microglial polarization via paracrine signaling, driving the transition from pro-inflammatory M1 microglia to anti-inflammatory M2 microglia, which subsequently secrete anti-inflammatory factors to support tissue repair (Soto and Howell 2014; Rathnasamy et al. 2019; Fu et al. 2023). MSCs have also been shown to modulate Müller glial cell activation via paracrine signaling. Activated Müller glial cells secrete neurotrophic and anti-inflammatory factors, thereby facilitating retinal repair. MSCs also suppress the excessive proliferation and fibrosis of Müller glial cells observed in retinal degenerative diseases, thereby mitigating neurodegeneration and inflammation in the retina (Tassoni et al. 2015; Forrester et al. 2020). MSCs facilitate the polarization of microglia from the pro-inflammatory M1 phenotype to the anti-inflammatory M2 phenotype by suppressing the HMGB1*,*TLR4 signaling pathway, effectively reducing retinal inflammation (Tong et al. 2024). MSCs preserve retinal structural integrity and attenuate neuronal apoptosis by suppressing Toll-like receptor 4 (TLR4) signaling, thereby downregulating pro-inflammatory factors and glial activation markers (Lejkowska et al. 2019).

### Resistance to oxidative stress in MSC

Oxidative stress is widely recognized as a critical driver of retinal cell damage and death in retinal degenerative diseases. In retinal injury models induced by oxidative stress, MSCs mitigate retinal damage by secreting antioxidant factors, which significantly reduce reactive oxygen species (ROS) levels and upregulate antioxidant enzyme activity (Tang et al. 2023). MSCs alleviate retinal oxidative stress by activating multiple antioxidant signaling pathways and modulating the expression of related genes. Studies demonstrate that MSCs activate the Nrf2 signaling pathway, enhancing cellular antioxidant defenses, reducing oxidative stress, and decelerating the progression of retinal damage and neurodegeneration (Sun et al. 2022; Bai and Wang 2024). Human Wharton’s jelly-derived MSCs (hWJ-MSCs) secrete erythropoietin (Epo) to counteract oxidative stress-induced retinal neuronal damage, markedly improve mitochondrial function, and enhance cell viability, collectively bolstering the antioxidant potential of MSCs (Shirley Ding et al. 2018).

### Anti-apoptotic effects of MSC

The anti-apoptotic effects of MSCs in retinal diseases extend beyond the secretion of growth and neuroprotective factors or the regulation of anti-inflammatory and immune responses. They are also intricately linked to the precise modulation of apoptosis-related gene expression and apoptotic signaling pathways (Chen et al. 2013; Jiang et al. 2014). MSCs inhibit apoptosis in retinal neurons by activating multiple signaling pathways, such as Akt, and suppressing the expression of apoptosis-related proteins, including cell cycle inhibitors (e.g., p53) and apoptotic effector molecules (e.g., Caspase-3) (Wang et al. 2024a).

### The vascular remodeling effect of MSC

Neovascularization plays a critical role in sustaining normal retinal function. However, pathological blood vessels are characterized by structural abnormalities, including thin walls and deficient tight junctions, disrupting retinal vascular homeostasis. A hallmark feature of diabetic retinopathy is retinal vascular abnormalities, encompassing vascular leakage, microvascular occlusion, microaneurysm formation, retinal edema, and pathological neovascularization (Zhao et al. 2024). Wet AMD is defined by choroidal neovascularization, a pathological hallmark that disrupts retinal structure and precipitates rapid vision deterioration (Ramkumar et al. 2010). MSCs secrete a dual repertoire of pro-angiogenic and anti-angiogenic factors, with their paracrine effects modulated by the retinal microenvironment and specific pathological contexts. MSCs facilitate physiological vascular development in the early stages of retinal diseases by fine-tuning the composition of secreted factors, while in advanced stages, they suppress pathological neovascularization to preserve retinal vascular homeostasis. Research indicates that MSC administration elevates intraocular levels of the anti-angiogenic factor TSP-1 in diabetic mice, effectively suppressing pathological neovascularization and mitigating vascular hyperpermeability (Ezquer et al. 2016). MSCs strengthen endothelial tight junctions, restoring vascular barrier integrity and alleviating retinal edema and hemorrhage. It’s reported that conditioned medium from amniotic MSCs markedly stimulates human umbilical vein endothelial cells proliferation, migration, and tube formation, while suppressing pathological neovascularization in a mouse model of retinal disease (Kim et al. 2016). MSCs exhibit the capacity to differentiate into pericyte-like cells, thereby stabilizing retinal microvasculature and preventing vascular regression (Mannino et al. 2020). Recent research reveals that 7S,14R-dihydroxy-docosahexaenoic acid (7S,14R-diHDHA) enhances MSC secretion of VEGF and HGF, markedly augmenting pericyte density in the retina and ameliorating diabetes-induced pericyte loss (Lu et al. 2024).

### MSC rescues retinal cell death caused by mitochondrial damage

Recent evidence highlights that MSCs facilitate bioenergetic crosstalk with retinal cells through mitochondrial transfer, thereby restoring impaired cellular energy metabolism. This mechanism offers a novel therapeutic strategy for leveraging MSCs in retinal degenerative diseases. It's demonstrated that MSCs transfer functional mitochondria to RPE cells and photoreceptors via intercellular nanotubes, thereby enhancing mitochondrial function, mitigating reactive oxygen species (ROS) levels, and promoting cell survival (Jiang et al. 2020). A 2019 study investigated the protective effects of MSC-mediated mitochondrial transfer on RGCs against mitochondrial complex I deficiency-induced neurodegenerative damage. In a mouse model, iPSC-derived MSCs (iPSC-MSCs) markedly enhanced RGC survival by improving energy metabolism and cellular viability, while attenuating retinal inflammation and decelerating the progression of retinal degeneration (Jiang et al. 2019). Recent studies reveal that BMSCs transfer mitochondria to retinal cells, particularly Müller glial cells, to restore mitochondrial functionality and enhance cell survival (Huang et al. 2024). Mitochondria transferred by BMSCs integrate with those of damaged cells, restoring energy production and mitigating neurodegenerative damage. This process facilitates damaged cell repair while suppressing glial activation, limiting glial proliferation and fibrosis, and alleviating retinal inflammation. These mechanisms highlight the potential of MSC-mediated mitochondrial transfer as an innovative approach for stem cell-based therapies targeting retinal degenerative diseases.

## The therapeutic effects of mesenchymal stem cells in animal models of retinal degenerative diseases

As the understanding of the pathological mechanisms underlying retinal degenerative diseases deepens, an increasing number of mouse models have been developed to effectively replicate human retinal degenerative conditions. These models provide valuable tools for investigating the mechanisms by which mesenchymal stem cells (MSCs) may treat retinal degenerative diseases, thereby advancing related research (Table 1).

**Table 1:** Application and mechanism of action of MSCs in retinal degenerative diseases

Disease	Animal model	MSCs type	Administration method	Mechanism of action	Refs.
Glaucoma	Chronic ocular hypertension rat model	Human umbilical cord MSCs	Intravitreal injection	Inhibits apoptotic pathways and reduces RGC loss	Wang et al. (2021)
Glaucoma	Laser-induced high intraocular pressure rat model	Rat bone marrow mesenchymal stem cells	Intravenous injection/intravitreal injection	Neuroprotection and modulation of the inflammatory microenvironment	Johnson et al. (2010)
Age-related macular degeneration	Light-induced retinal damage rat model	Rat bone marrow mesenchymal stem cell	Intravitreal injection	Inhibition of photoreceptor death and neuroprotection	Zhang and Wang (2010), Xu et al. (2014)
Age-related macular degeneration	Light-induced retinal damage mouse model	Adipose-derived stem cells	Intravitreal injection	Neuroprotection and inhibition of apoptosis	Sugitani et al. (2013), Tsuruma et al. (2014)
Age-related macular degeneration	Degradation model of photoreceptors and RPE induced by sodium iodate	Rat bone marrow mesenchymal stem cells	Intravitreal injection	Protects cone cells and improves visual function	Lam et al. (2021)
Retinitis pigmentosa	Rhodopsin knockout mouse model	Rat bone marrow mesenchymal stem cells	Intravitreal injections	Extended photoreceptors survival	Arnhold et al. (2007)
Retinitis pigmentosa	RPGR knockout mouse model	Mesenchymal stem cell from human exfoliated deciduous teeth	Subretinal injection	Improvement of retinal microenvironment and protection of photoreceptors	Li et al. (2019)
Retinitis pigmentosa	RCS rat model	Human umbilical cord mesenchymal stem cells	Intravenous injection	Delaying retinal degeneration	Liang et al. (2022b)
Diabetic retinal degeneration	STZ-induced diabetic retinopathy rat l	Human umbilical cord mesenchymal stem cells	Intravitreal injection	Reduce inflammation and protect vascular function	Zhao et al. (2020)
Diabetic retinal degeneration	STZ-induced diabetic retinopathy rat model	Rat bone marrow mesenchymal stem cells	Intravitreal injection	Protection of retinal structure and vascular function	Abdel-Kawi and Hashem (2022)
Diabetic retinal degeneration	STZ-induced diabetic retinopathy rat model	Human umbilical cord mesenchymal stem cells	Subconjunctival injection	Reduce mitochondrion division, apoptosis, and neuroprotective cells and blood vessels	Jo et al. (2023)

### MSC application in glaucoma animal models

Glaucoma models encompass various methods such as intraocular microbead injection, laser photocoagulation, scleral vein cauterization, optic nerve compression, hyaluronic acid injection, and genetically engineered rodent models, all characterized by RGC damage (Overby and Clark 2015; Biswas and Wan 2019). Human umbilical cord-derived mesenchymal stem cells demonstrate significant survival and migratory capacity in a chronic ocular hypertension (COH) rat model of glaucoma. Following intravitreal injection, hUC-MSCs migrate to the injured retinal regions, where they mitigate RGC loss by inhibiting the Caspase-8-dependent apoptotic pathway, ultimately alleviating retinal damage (Wang et al. 2021). Adipose-derived stem cells exhibit significant potential in reducing apoptosis and facilitating RGC regeneration. In retinal hypoxia models, ADSCs exert their effects by secreting anti-apoptotic factors such as TIMP-1, TIMP-2, and osteoprotegerin, along with pro-proliferative molecules, collectively enhancing RGC survival (Dov et al. 2023). In laser-induced glaucoma models, MSCs reduce the apoptosis of retinal ganglion cells by secreting neurotrophic factors such as brain-derived neurotrophic factor and glial cell line-derived neurotrophic factor, and by modulating the inflammatory microenvironment and intravitreal injection of MSCs shows better efficacy than systemic administration (Johnson et al. 2010).

### MSC application in AMD animal models

Excessive light exposure contributes to photoreceptor degeneration and retinal pigment epithelial cell damage and has been strongly linked to the development of age-related macular degeneration. Light-induced retinal damage models in mice have been widely employed to study age-related macular degeneration and are characterized by inflammation and infiltration of microglial cells (Organisciak et al. 1996; Rutar et al. 2011). MSC transplantation has emerged as a promising therapeutic approach for light-induced retinal damage, supported by accumulating evidence of its effectiveness. Subretinal transplantation of bone marrow-derived mesenchymal stem cells in light-damaged rat models has been shown to inhibit photoreceptor apoptosis, slow retinal degeneration, and provide neuroprotection by secreting BDNF and bFGF (Zhang and Wang 2010). Systemic administration of mesenchymal stem cells has also demonstrated the ability to prevent photoreceptor apoptosis and confer neuroprotection in preclinical studies (Xu et al. 2014). Adipose-derived mesenchymal stem cells have exhibited comparable neuroprotective effects in age-related macular degeneration models, further supporting the therapeutic potential of MSCs in retinal disease treatment (Sugitani et al. 2013; Tsuruma et al. 2014).

Sodium iodate, a chemical widely used to model age-related macular degeneration, causes retinal pigment epithelial cell degeneration through different delivery routes. Systemic injection leads to widespread RPE degeneration, subretinal injection induces localized loss of RPE and photoreceptors, and intravitreal injection primarily triggers photoreceptor degeneration (Kim and Qian 2022). In retinal pigment epithelial cell injury models induced by sodium iodate, co-culture with mesenchymal stem cells significantly enhances RPE cell survival and suppresses apoptosis by downregulating NF-κB and NLRP3 inflammasome activation (Mao et al. 2018). In sodium iodate-induced retinal degeneration models, the combined intravenous and subretinal delivery of human dental pulp stem cells markedly enhances visual function and provides robust protection for cone cells (Lam et al. 2021).

### MSC application in RP animal models

Retinitis pigmentosa is a group of genetic degenerative disorders marked by the progressive dysfunction and loss of photoreceptors and retinal pigment epithelial cells, ultimately resulting in severe vision impairment or blindness. To explore the underlying mechanisms and therapeutic strategies for retinitis pigmentosa, researchers have developed a wide array of animal models, including naturally occurring genetic models. For instance, rd mice display photoreceptor degeneration driven by a nonsense mutation in the *Pde6b* gene, RCS rats experience RPE dysfunction due to a mutation in the *Mertk* gene, and rds mice exhibit disrupted outer segment disk formation caused by mutations in the *Peripherin,rds* gene (Qin et al. 2023). Transgenic models include mice with targeted knockouts of the *Rhodopsin*, *Rpgr*, and *Rpe65* genes. Chemically induced models, such as N-methyl-N-nitrosourea (MNU)-induced photoreceptor apoptosis models, have also been extensively employed in retinitis pigmentosa research (Gopalakrishnan et al. 2023; Yang et al. 2024a). These models recapitulate the genetic and pathological characteristics of human retinitis pigmentosa, serving as indispensable tools for uncovering its underlying mechanisms and advancing therapeutic development.

In 2007, researchers demonstrated that subretinal transplantation of BMSC into rhodopsin-knockout mice significantly reduced photoreceptor apoptosis. The MSCs exerted paracrine effects and engaged in partial cellular fusion, creating a more favorable environment for photoreceptor survival (Arnhold et al. 2007). Subsequent studies revealed that mesenchymal stem cells derived from umbilical cord and dental pulp enhanced the retinal microenvironment, safeguarded photoreceptors, and contributed to partial restoration of visual function (Li et al. 2019; Liang et al. 2022b). Retinal progenitor cells derived from primitive human mesenchymal stem cells have shown significant promise in treating retinal degenerative diseases. These cells enhanced photoreceptor survival through the secretion of neurotrophic factors, activation of neurogenesis and neuroprotective genes, and modulation of multiple signaling pathways, presenting novel perspectives for cell-based therapies in retinitis pigmentosa (Brown et al. 2022).

### MSC application in DR animal models

Diabetes represents the fastest-growing metabolic disorder globally, with diabetic retinopathy being among the most prevalent and debilitating complications driven by chronic hyperglycemia (Cole and Florez 2020). Current therapeutic strategies for diabetic retinopathy are limited, positioning mesenchymal stem cell-based therapies as a promising and innovative approach for addressing diabetes and its complications (Liu et al. 2022b). Streptozotocin (STZ), widely used for establishing diabetic retinopathy models, induces diabetes by selectively destroying pancreatic B cells, impairing insulin synthesis and secretion, and triggering persistent hyperglycemia. Hyperglycemia induces the loss of retinal ganglion cells and triggers pathological alterations in the retinal microvasculature (Yang et al. 2009). Genetically predisposed mouse models, such as db*/*db mice, closely resemble the genetic and pathological characteristics of human diabetic retinopathy, serving as essential tools for exploring disease mechanisms and advancing treatments.

MSC transplantation mitigates streptozotocin-induced diabetic retinopathy in rats by modulating cytokine secretion, attenuating retinal damage and apoptosis, suppressing aberrant angiogenesis, and alleviating inflammation (Fiori et al. 2018). In streptozotocin-induced diabetic rats, human umbilical cord-derived mesenchymal stem cells ameliorate inflammation by suppressing MIAT expression, reducing pro-inflammatory factors such as interleukin-1 beta and interleukin-6, and enhancing tight junction protein expression (Yu et al. 2020). These molecular changes restore vascular barrier integrity, reduce retinal microvascular leakage, and slow disease progression. Moreover, the therapeutic potential of mesenchymal stem cells for diabetic retinopathy is significantly enhanced when combined with small-molecule drugs. The therapeutic potential of mesenchymal stem cells for diabetic retinopathy is significantly enhanced when combined with small-molecule drugs. In STZ-induced diabetic retinopathy models, preconditioning UC-MSCs with all-trans retinoic acid significantly enhanced their efficacy, resulting in more effective suppression of retinal inflammation and apoptosis, alongside marked improvements in microvascular function (Zhao et al. 2020). The co-administration of MSCs with melatonin synergistically enhanced therapeutic outcomes, notably reducing pathological neovascularization and microvascular leakage through the regulation of vascular-associated factors, including VEGF, apolipoprotein A1, and retinol-binding protein 4. This further safeguarded retinal structural and functional integrity (Abdel-Kawi and Hashem 2022). High glucose levels exacerbate oxidative stress and mitochondrial fragmentation, which significantly hinder MSC therapeutic efficacy. Tacrolimus, by suppressing dynamin-related protein 1 activation, mitigates mitochondrial fragmentation and apoptosis, thereby amplifying the protective effects of MSCs in diabetic retinopathy models (Jo et al. 2023). Tacrolimus-preconditioned MSCs exhibited significantly greater efficacy compared to untreated MSCs in restoring retinal neurovascular integrity and function in diabetic retinopathy models.

## The role of mesenchymal stem cell-derived exosomes in retinal degenerative diseases

**Fig. 5:**
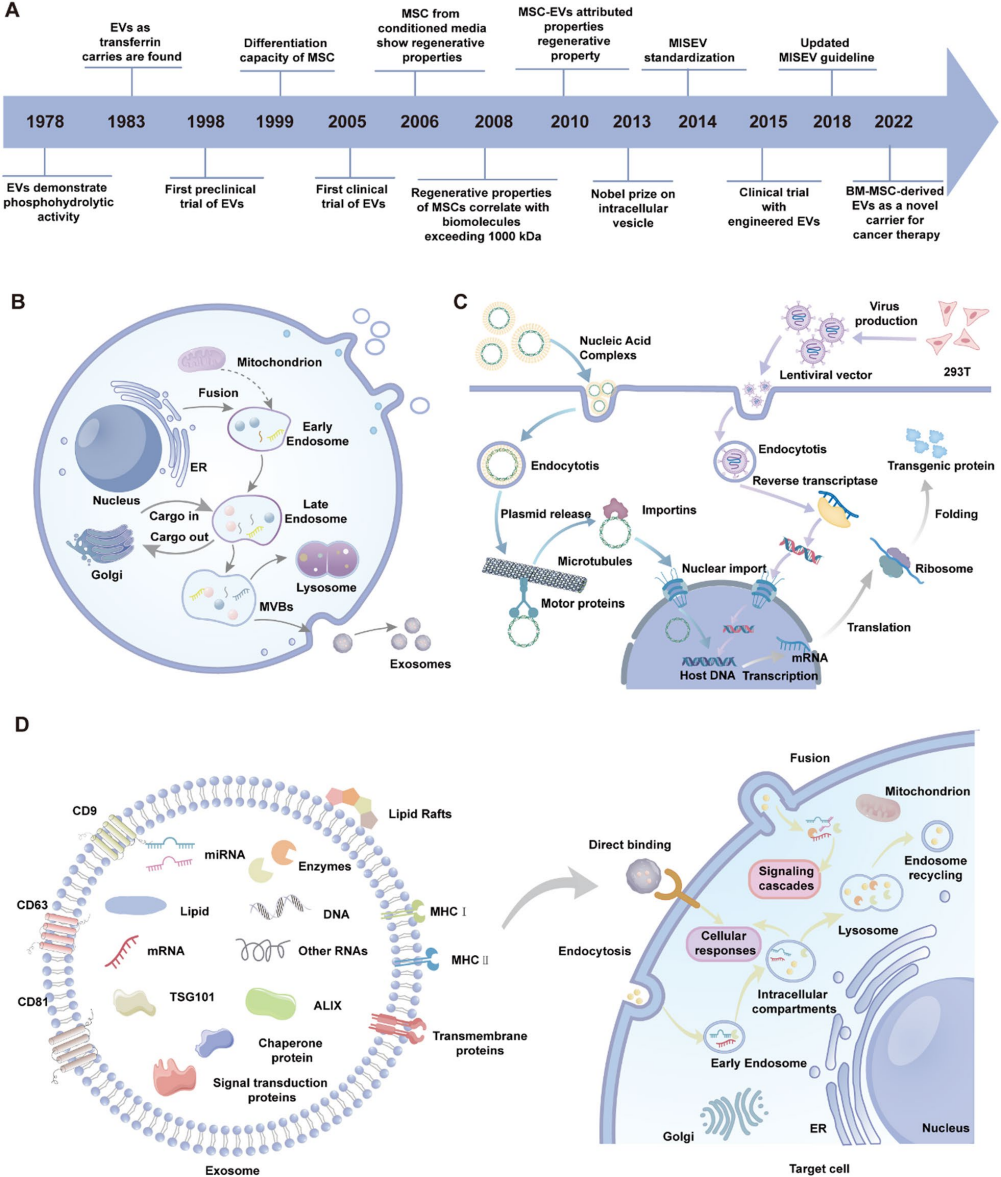
Timeline, biogenesis, therapeutic production, and functional mechanisms of MSC-derived Evs. **A** Timeline of research progress on Extracellular vesicles (EVs). **B** Mechanisms of exosome biogenesis. Exosomes are formed via the endosomal pathway, involving early endosomes, late endosomes, multivesicular bodies (MVBs), and lysosomes, before being secreted as exosomes. **C** Production and delivery mechanisms of therapeutic EVs. Engineered EVs are loaded with nucleic acids and proteins using lentiviral vectors or plasmid systems. **D** Structural composition and functional mechanisms of exosomes. Exosomes are enriched with transmembrane proteins (e.g., CD9, CD63, CD81), nucleic acids (DNA, mRNA, miRNA), lipids, and enzymes. These molecules achieve functional delivery by binding to target cells, triggering signaling cascades, or fusing with intracellular structures

Exosomes, as essential mediators of intercellular communication, have emerged as a pivotal focus in therapeutic strategies for retinal degenerative diseases (Table 2). Mesenchymal stem cells secrete a variety of extracellular vesicles, including exosomes, a specialized subtype measuring 30 to 150 nm, which can efficiently cross the blood-retinal barrier and precisely target diseased regions. From the first discovery of extracellular vesicles with phosphate hydrolysing activity in 1978 to their engineering modification for cancer therapy in recent years, research has deepened and made significant progress (Fig. 5A).

**Table 2:** Application of mesenchymal stem cell exosomes in the treatment of retinal degenerative diseases

Disease	Animal model	Exosomes (EVs) type	Administration method	Biological effect	Refs.
Glaucoma	In-vitro oxygen–glucose deprivation rat model	Human bone marrow mesenchymal cell derived EVs	Intravitreal injection	MSC-EXO can persist in RGCs for up to 14 days and provide neuroprotection by reducing cell apoptosis and inflammation	Mathew et al. (2019)
Glaucoma	Optic nerve crush mouse model	Human embryonic stem cell derived EVs	Intravenous injection	ES-MSC-EVs significantly enhance RGC survival, protect optic nerve axons, improve vision-related behaviors, and prevent the degeneration and thinning of the retinal nerve fiber layer	Seyedrazizadeh et al. (2020)
Retinitis pigmentosa	MNU-induced photoreceptor loss mouse model	Human bone marrow mesenchymal cell derived exosomes	Intravitreal injection	MSC-EXOs target Pdcd4 via exosomal miR-21, significantly enhancing the resistance of photoreceptor cells to apoptosis while preserving retinal structure and function	Deng et al. (2021)
Retinitis pigmentosa	Retinitis pigmentosa rd10 mouse model	Human umbilical cord MSCs exosomes	Intravitreal injection	miR-126 in MSC-Exos downregulates the HMGB1 signaling pathway and suppresses inflammation in diabetic rats	Zhang et al. (2022)
Retinitis pigmentosa	Retinitis pigmentosa RCS rat	Rat bone marrow mesenchymal cell derived exosomes	Intravitreal injection	IFNγ enhances the neuroprotective effects of MSC-derived exosomes on degenerative retinal photoreceptors, potentially by modulating microglial activity to reduce inflammation and alleviate immune-mediated retinal damage through tsRNA signaling	A et al. (2024)
Diabetic retinopathy	STZ-induced diabetic retinopathy rat model	Rat bone marrow mesenchymal cell derived exosomes	Intravitreal injections	Exosomes alleviate Wnt3a-triggered retinal damage, reducing oxidative stress, inflammation, and angiogenesis, ultimately restoring impaired retinal function	Ebrahim et al. (2022)
Diabetic retinopathy	STZ-induced diabetic retinopathy rat model	Human umbilical cord mesenchymal stem cell derived EVs	Intravitreal injections	Exosomes deliver miR-18b to inhibit the MAP3K1/NF-κB pathway, reducing inflammation, protecting photoreceptors, and improving vascular barrier function in DR mice	Xu et al. (2021)
Diabetic retinopathy	STZ-induced diabetic retinopathy rat model	Rat adipose mesenchymal stem cell derived EVs	Intravitreal injections	MSC-EVs transport miR-192 to alleviate inflammation and angiogenesis by targeting and negatively regulating ITGA1, thereby improving diabetic retinal damage	Gu et al. (2021)
Diabetic retinopathy	STZ-induced diabetic retinopathy rat model	Umbilical cord-derived mesenchymal stem cells-derived exosomes	Intravitreal injections	The expression of miR-126 in MSC-Exos protects the retina by downregulating the HMGB1 signaling pathway and reducing NLRP3 inflammasome activity	Zhang et al. (2019)
Diabetic retinopathy	STZ-induced diabetic retinopathy rat model	Bone marrow mesenchymal stem cells-induced exosome	Intravitreal injections	Up-regulation of miR-486-3p induced by BMSC-derived exosomes played a protective role in DR mice via TLR4/NF-κB axis repression	Li et al. (2021a)
Diabetic retinopathy	Oxygen-induced retinopathy mouse model	Human umbilical cord mesenchymal stem cell derived EVs	Intravitreal injections	PEDF-sEVs effectively enhanced the anti-angiogenic, anti-inflammatory, and neuroprotective effects of PEDF by increasing its stability and penetrability	Fan et al. (2023)
Diabetic retinopathy	STZ-induced diabetic retinopathy rat model	Human bone marrow mesenchymal cell derived exosomes	Intravitreal injections	EVs loading bevacizumab reduce the formation and inflammation of retinal neovascularization, prolong the effective period of the medicine and reduce the injection frequency	Reddy et al. (2023)

MSC-derived exosomes are released from multivesicular bodies via fusion with the plasma membrane, enabling their entry into the extracellular environment. Owing to the overlapping morphological and functional characteristics of different extracellular vesicles, exosomes are often collectively referred to as “extracellular vesicles in many studies” (Tian et al. 2023; Wu et al. 2024) (Fig. 5B). MSC-derived exosomes transport diverse bioactive molecules, facilitating intercellular communication to modulate immune responses, apoptosis, cellular differentiation, and tissue regeneration (Nicodemou et al. 2023). The lipid profile of MSC exosomes, including phospholipids and cholesterol, plays a pivotal role in preserving their structural stability and functional efficacy. Exosomal surface proteins such as CD9, CD63, and CD81 are key biomarkers for identification and functional characterization, while their cargo, including proteins, lipids, mRNA, and miRNA, plays a critical role in regulating gene expression and cellular activities in recipient cells (Morishita et al. 2017).

MSC exosome biogenesis entails the inward budding of endosomes to form multivesicular bodies, which subsequently fuse with the plasma membrane to release exosomes into the extracellular space (Ostrowski et al. 2010). MSC-derived exosomes mirror the biological functions of mesenchymal stem cells, demonstrating capacities for tissue regeneration, immune regulation, and anti-inflammatory activity. Additionally, MSC exosomes possess several advantageous characteristics, including nanoscale size, low immunogenicity, and high permeability, establishing them as promising candidates for the treatment of various human diseases (Rani et al. 2015; Luo et al. 2021).

The successful application of MSC-based therapies critically depends on the efficient isolation and purification of exosomes, as these methods significantly influence their yield and purity. Common purification techniques include differential ultracentrifugation, ultrafiltration, size-exclusion chromatography, polyethylene glycol precipitation, and magnetic bead-based immunoprecipitation. Differential ultracentrifugation remains the most widely adopted method in laboratory settings, attributed to its operational simplicity, high efficiency, and the ability to achieve relatively high purity (Livshits et al. 2015).

Engineering MSC-derived exosomes via physical, chemical, or biological modifications significantly improves their therapeutic efficacy (Fig. 5C). For example, genetic editing or chemical modification allows MSC exosomes to deliver specific miRNAs, siRNAs, or gene-regulating molecules to target critical signaling pathways in retinal pathologies (Liu et al. 2022c). These functional enhancements make engineered MSC-derived exosomes highly promising candidates for retinal repair and regeneration, offering significant therapeutic potential.

The therapeutic potential of MSC-derived exosomes in treating retinal degenerative diseases is mediated through multiple synergistic mechanisms (Wang et al. 2024b). Specific ligands present on MSC-derived exosome membranes interact with receptors on target cells, triggering intracellular signaling cascades that regulate proliferation, differentiation, and metabolic processes. Upon fusing with the membranes of target cells, MSC-derived exosomes release their cargo of bioactive molecules, modulating gene expression to enhance cellular repair and tissue regeneration. Furthermore, MSC-derived exosomes secrete small RNAs and signaling molecules that directly bind to surface receptors on target cells, fine-tuning immune responses, suppressing inflammation, and restoring tissue functionality (Fig. 5D).

**Fig. 6:**
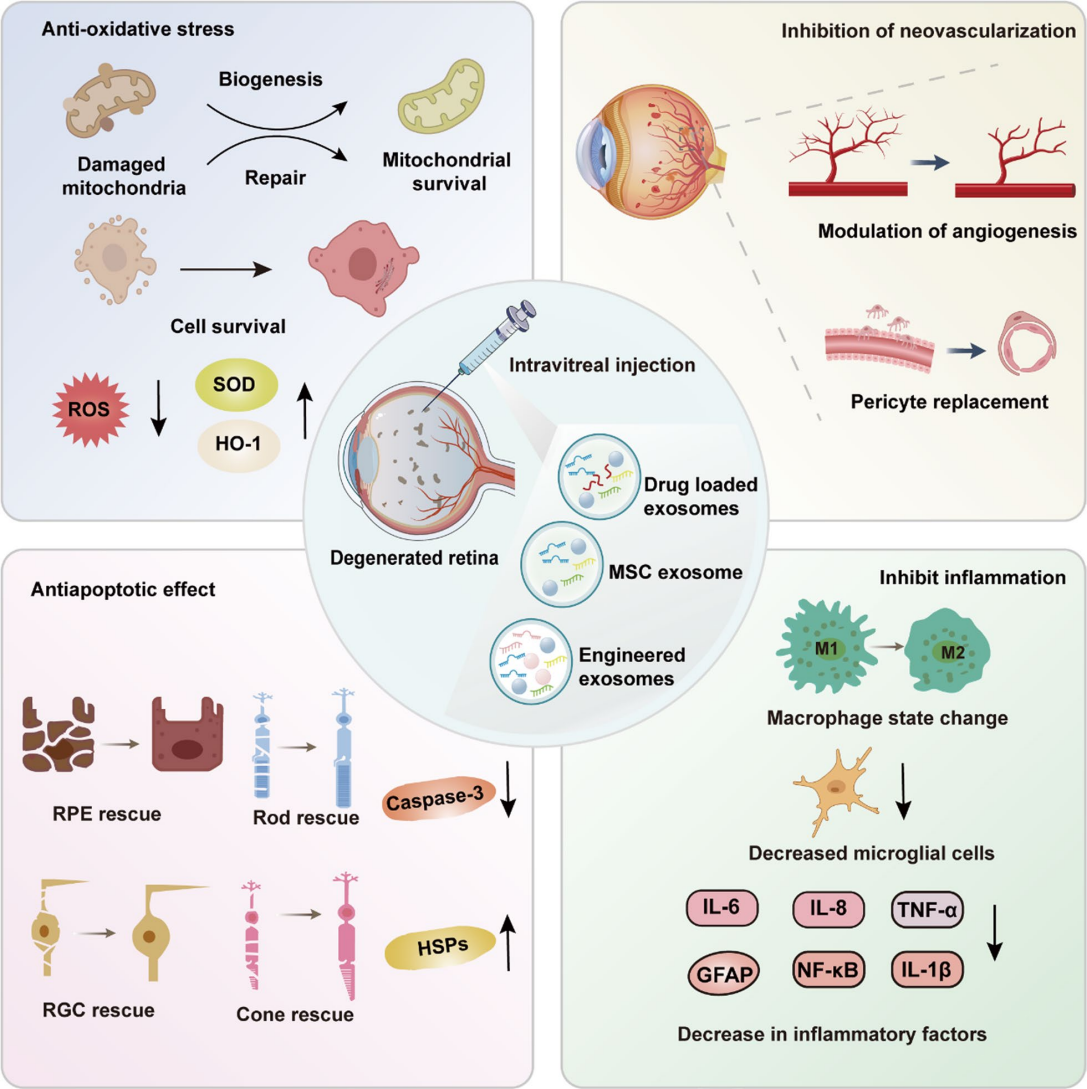
Therapeutic mechanisms of MSC-derived and engineered exosomes in retinal degeneration. Reduces oxidative stress by lowering ROS levels and increasing SOD and HO-1 expression to support mitochondrial repair and survival. Inhibits neovascularization by modulating angiogenesis and restoring vascular integrity through pericyte replacement. Exhibits anti-apoptotic effects by reducing Caspase-3 activity and enhancing HSPs to protect RPEs, photoreceptors, and retinal ganglion cells (RGCs). Suppresses inflammation by shifting macrophages from the M1 to M2 state, decreasing microglial activation, and downregulating inflammatory markers (IL-6, TNF-α, NF-κB). These pathways collectively support retinal protection and regeneration

Experimental studies have highlighted the remarkable therapeutic potential of MSC-derived exosomes in preclinical models of retinal degenerative diseases. These exosomes exert multifaceted effects positioning them as a promising strategy for retinal disease treatment (Fig. 6).

### Therapeutic effects in glaucoma models

In glaucoma models, MSC-derived exosomes have shown clear neuroprotective effects on RGCs, offering new therapeutic possibilities. Research revealed that human bone marrow MSC exosomes exosomes significantly alleviated ischemic damage in a rat model of retinal ischemia–reperfusion injury (Mathew et al. 2019). MSC-derived were efficiently internalized by retinal neurons, RGCs, and microglial cells, where they conferred neuroprotection by mitigating apoptosis and suppressing inflammation. Additionally, they further confirmed that MSC-Exo could persist in RGCs for up to 14 days without inducing retinal toxicity, offering critical insights for clinical translation (Mathew et al. 2021).

Exosomes derived from embryonic stem cell-derived MSCs highly enhanced RGC survival, safeguarded optic nerve axons, improved vision-associated behaviors, and prevented retinal nerve fiber layer thinning (Seyedrazizadeh et al. 2020). It's reported that small extracellular vesicles derived from bone marrow mesenchymal stem cells significantly preserved retinal ganglion cell populations and mitigated axonal degeneration in 6-month-old genetic glaucoma mouse models. However, in 9-month-old glaucoma mouse models, bone marrow MSC-derived small EVs exhibited limited therapeutic benefits, indicating that the efficacy of unmodified MSC-derived exosomes may decrease with disease progression (Mead et al. 2018).

### Therapeutic effects in retinitis pigmentosa models

MSC-Exo exhibit notable protective effects on retinal photoreceptors. It was reported that MSC-Exos inhibited photoreceptor apoptosis in a mouse model of *N*-methyl-*N*-nitrosourea-induced retinal injury. Through the delivery of abundant miR-21, MSC-Exos targeted programmed cell death 4 (Pdcd4), substantially improving photoreceptor resistance to apoptosis while preserving retinal structure and function (Deng et al. 2021). In a laser-induced retinal injury model, MSC-derived Exosomes alleviated inflammation by downregulating monocyte chemoattractant protein-1 (MCP-1), reducing macrophage recruitment and infiltration, and consequently facilitating retinal functional recovery (Yu et al. 2016). Additionally, MSC-derived extracellular vesicles demonstrated photoreceptor protection in a retinitis pigmentosa mouse model by mitigating inflammation-induced cell death through the miR-146a-Nr4a3 signaling pathway (Zhang et al. 2022). A recent study demonstrated that interferon-γ-stimulated MSC-derived exosomes effectively protected retinal photoreceptor cells by modulating immune responses, significantly reducing photoreceptor apoptosis in RCS rat models (A et al. 2024). These Exosomes not only promoted retinal cell survival but also alleviated immune-mediated retinal damage by suppressing excessive microglial activation and inflammation through the modulation of tRNA-derived small RNAs (tsRNAs). These results suggest that MSC-derived exosomes offer innovative therapeutic strategies for RP through the bioactive molecules they contain.

### Therapeutic effects in diabetic retinopathy models

In DR models, MSC-derived Exos exhibited dual therapeutic benefits, effectively mitigating inflammation and photoreceptor apoptosis while enhancing vascular function and regulating the retinal microenvironment. Fundus fluorescein angiography (FFA) and optical coherence tomography (OCT) revealed that MSC-Exo effectively prevented early-stage vascular damage and retinal structural deterioration in diabetic retinas (Fu et al. 2021). Human umbilical cord MSC-EVs reduced inflammation and safeguarded photoreceptors in diabetic retinopathy by delivering miR-18b, which inhibited the MAP3K1, NF-κB pathway and enhanced vascular barrier integrity (Xu et al. 2021). Bone marrow MSC-Exos attenuated oxidative stress and inflammation in diabetic retinopathy by inhibiting the Wnt, β-catenin pathway, upregulating superoxide dismutase (SOD), and suppressing VEGF-driven pathological neovascularization (Ebrahim et al. 2022). MSC-Exos targeted integrin α1 (ITGA1) via the delivery of microRNA-192, leading to a significant reduction in angiogenesis-associated factors, including VEGFA and CD31, and mitigating Müller cell activation in diabetic retinopathy (Gu et al. 2021). MSC-Exos effectively inhibited hyperglycemia-induced abnormal proliferation of retinal microvascular endothelial cells in both in vivo and in vitro studies (Cao et al. 2021; Gu et al. 2021). Additional miRNAs contained in MSC-Exos have been shown to regulate multiple critical retinal pathways, suppressing inflammatory neovascularization and facilitating the restoration of retinal structure and function (Zhang et al. 2019; Li et al. 2021a, b; He et al. 2022; Liang et al. 2022a; Sun et al. 2024).

Exosome therapy for DR is not limited to miRNA-mediated signaling. In protein-mediated regulation, MSC-EVs delivered NEDD4 protein to promote PTEN ubiquitination and degradation, activating the AKT, NRF2 pathway to reduce ROS levels, upregulate anti-apoptotic proteins, and alleviate retinal oxidative stress and apoptosis (Sun et al. 2022).

MSC-EVs have been investigated as drug delivery platforms to enhance and extend the efficacy of anti-VEGF therapies. For example, bevacizumab-loaded MSC-EVs outperformed bevacizumab alone in diabetic retinopathy rat models by reducing retinal neovascularization and inflammation, prolonging therapeutic duration, lowering injection frequency, and ultimately alleviating patient burden (Reddy et al. 2023).

## Application of MSC in clinical trials of retinal diseases

Mesenchymal stem cell transplantation has emerged as a promising therapeutic approach for retinal degenerative diseases, with significant advancements in preclinical research and encouraging findings in clinical trials (Table 3). Early-phase clinical studies have highlighted the favorable safety profile of MSC-based therapies for retinal degenerative diseases, with notable improvements in visual function reported in certain patients. In 2016, researchers initiated a Phase I safety trial in 11 patients with advanced retinitis pigmentosa, utilizing subretinal transplantation of autologous adipose-derived MSCs (Oner et al. 2016). Over the follow-up period, one patient experienced significant visual improvement, while three others reported enhanced light and color perception. However, ocular complications such as choroidal neovascularization and epiretinal membranes occurred in six patients, underscoring the need to address local adverse effects and variability in therapeutic efficacy despite the short-term safety of this approach.

**Table 3:** Clinical trials of mesenchymal stem cells in the treatment of retinal degenerative diseases were registered in ClinicalTrials.gov

Study title	First posted time	ClinicalTrials.gov identifier	Phase	Disease	Injection medication	Sponsor
Management of retinitis pigmentosa by mesenchymal stem sells by Wharton’s jelly derived mesenchymal stem cells (WJ-MSC)	2019-01-28	NCT04224207	Phase 3	Retinitis pigmentosa and inherited retinal dystrophy	WJ-MSC	Ankara Universitesi Teknokent
Intravitreal injection of MSCs in retinitis pigmentosa	2012-02-10	NCT01531348	Phase 1	Retinitis pigmentosa	BM-MSC	Mahidol University
Safety of cultured allogeneic adult umbilical cord derived mesenchymal stem cells for eye diseases	2021-12-07	NCT05147701	Phase 1	Diabetic retinopathy and retinitis pigmentosa	UC-MSC	The Foundation for Orthopaedics and Regenerative Medicine
Role of UC-MSC and CM to inhibit vision Loss in Retinitis Pigmentosa	2023-06-18	NCT05909488	Phase 2/3	Retinitis pigmentosa	UC-MSC and conditioned medium	PT. Prodia Stem Cell Indonesia
The effect of stem cells and stem cell exosomes on visual functions in patients with retinitis pigmentosa	2022-06-09	NCT05413148	Phase 2/3	Retinitis Pigmentosa	WJ-MSC	TC Erciyes University
Intravitreal mesenchymal stem cell transplantation in advanced glaucoma	2014-01	NCT02330978	Phase 1	Glaucoma	BM-MSC	University of Sao Paulo

In 2017, researchers conducted a non-randomized, open-label, single-center study in 17 patients with diabetic retinopathy who underwent intravenous transplantation of autologous bone marrow-derived MSCs (Gu et al. 2018). The study revealed that autologous bone marrow MSC transplantation significantly lowered fasting blood glucose and high-sensitivity C-reactive protein (hs-CRP) levels. Among patients with severe non-proliferative DR, reductions in central macular thickness and improvements in best-corrected visual acuity (BCVA) were observed, while no notable effects were detected in those with non-high-risk proliferative DR. Among patients with severe non-proliferative DR, reductions in central macular thickness and improvements in best-corrected visual acuity were observed, while no notable effects were detected in those with non-high-risk proliferative DR. These results suggest that BMSC transplantation not only has a better safety profile, but also demonstrates potential therapeutic benefits in DR treatment.

In a separate Phase I clinical trial, intravitreal injections of autologous bone marrow MSCs were administered to RP patients, with follow-up extending over seven years (Tuekprakhon et al. 2021). During the 12-month follow-up, minor and transient adverse events were observed, accompanied by statistically significant improvements in BCVA. These adverse events resolved and returned to baseline by the end of the follow-up period. Nonetheless, one patient experienced a serious but manageable adverse event in year 3, suggesting that the treatment still needs to be further optimised in terms of safety and efficacy.

A Phase III clinical trial of 32 RP patients receiving WJ-MSC transplantation revealed a strong safety profile and therapeutic efficacy. Follow-up evaluations at 6 and 12 months showed significant improvements in retinal structure, BCVA, and visual field indices (Özmert and Arslan 2020a, b). Clinical findings have demonstrated that WJ-MSC transplantation is both safe and effective. During follow-up, significant improvements were observed in retinal structure, best-corrected visual acuity, and visual field index at 6 months, with these benefits persisting at 12 months. These results further substantiate the potential of MSC therapy as a treatment for RP patients.

Recent clinical trials have highlighted the efficacy of subchoroidal and intravenous transplantation of allogeneic MSCs, demonstrating substantial improvements in visual function with no severe systemic or ocular complications reported (Kahraman and Oner 2020; Zhao et al. 2021). These findings underscore the therapeutic potential of MSCs in restoring vision. Adherence to clinical reporting guidelines and international consensus, particularly with regard to route of administration, dosage and source, will further promote MSC as an effective treatment for retinal degenerative diseases (Renesme et al. 2025).

## MSC delivery strategies for retinal degenerative diseases

In MSC therapy for the treatment of retinal degenerative diseases, the selection of an appropriate delivery strategy is crucial for efficacy. Different delivery routes have a significant impact on stem cell survival, distribution, and ultimately therapeutic efficacy. Therefore, the selection of more efficient and safe delivery routes is an important step to promote the clinical application of MSC for retinal diseases.

Stem cell delivery can be mainly categorized into local delivery and systemic delivery. Systemic delivery is valued for its less invasive nature. Although systemic delivery can avoid local trauma, it cannot ensure the precise distribution of stem cells in the eye. Local delivery, on the other hand, can ensure high concentration accumulation of cells in the target area, but delivery of high doses of cells may accelerate cell death (Andrzejewska et al. 2021). Therefore, when choosing a delivery method, a balance needs to be struck between effectiveness, trauma, and safety.

Subconjunctival and intravenous injections are systemic delivery routes. Subconjunctival injection is performed by injecting MSC under the conjunctiva, where the stem cells gradually diffuse into the eye through the eye’s natural barrier. This method is minimally invasive and simple to perform, and is suitable for early or mild retinopathy. Nevertheless, the disadvantage of subconjunctival injection is that it is difficult for the stem cells to penetrate the retina effectively, resulting in a limited and short-term therapeutic effect (Cassano et al. 2023). Intravenous injection of stem cells to the eye through the bloodstream is simple and avoids local trauma; however, MSCs rarely reach the eye and may be carried by the bloodstream to other sites, resulting in adverse effects and a lack of therapeutic efficacy (Abd Rashid et al. 2022).

In contrast, intravitreal localized delivery is dominant in the treatment of retinal diseases due to its higher efficiency (Holan et al. 2021). Intravitreal injection is one of the commonly used local delivery modalities. By injecting MSC into the vitreous cavity, stem cells are able to rapidly reach the retina. Intravitreal injections are relatively easy to perform and can be done under local anesthesia with minimal trauma. However, intravitreal injections do not ensure that the stem cells reach the lesion precisely and the therapeutic effect is limited (Hussain et al. 2019).

Subretinal injections, on the other hand, inject stem cells directly into the space underneath the retina, allowing the cells to reach the exact area of damage. This route has strong therapeutic benefits, especially for diseases that require precise retinal repair (Scruggs et al. 2019). However, subretinal injections require surgery, which is more invasive and can lead to complications such as retinal damage, hemorrhage or detachment, making them relatively risky.

Subchoroidal injections are mainly used to treat retinal diseases such as macular degeneration. By delivering stem cells directly to the subchoroid, subchoroidal injection can effectively improve the function of the subretinal layer, especially repairing the retinal pigment epithelial cells. However, this method requires high-precision surgical skills and carries the risk of choroidal injury during the operation, limiting its clinical application (Li et al. 2023a).

## Application challenges of MSC-based therapies for retinal degenerative diseases

MSCs renowned for their robust immunomodulatory, anti-inflammatory, anti-apoptotic, differentiation, and regenerative properties, have unlocked multiple therapeutic avenues, providing diverse intervention strategies for retinal degenerative diseases. The versatility of MSCs underscores their therapeutic potential and enables the restoration of retinal function by leveraging their exceptional self-renewal and multipotent differentiation properties. Their low immunogenicity minimizes the risk of immune rejection in allogeneic transplantation. Moreover, MSCs can be harvested from adult tissues, circumventing the ethical concerns. The widespread availability and ease of isolation of MSCs further enhance their feasibility for clinical translation. Furthermore, engineering advancements, including genetic modification and integration with biomaterials, have further optimized MSC functionality, opening new avenues for addressing complex diseases (Ozawa et al. 2008). These attributes establish MSCs as a multifaceted and innovative therapeutic platform for retinal disease treatment, underscoring their significant potential in clinical applications.

**Fig. 7:**
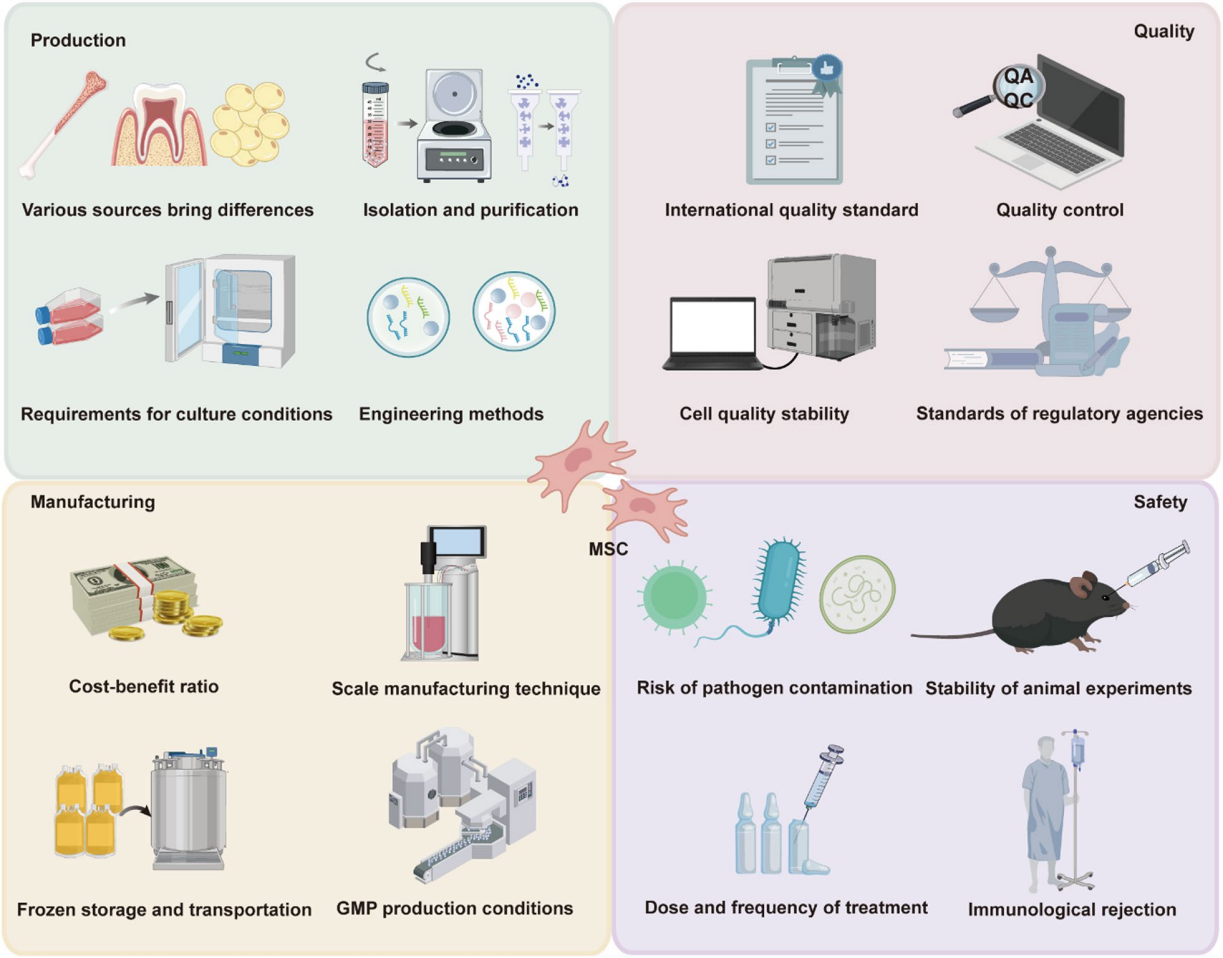
Challenges in the Clinical Application of MSC. Addressing these challenges is critical for the successful clinical translation of MSC-based therapies. Variability in cell sources, isolation methods, culture conditions, and engineering techniques, which affect cell quality and consistency. The need for scalable, cost-effective production methods, GMP compliance, and reliable storage and transportation systems. Adherence to international standards, robust quality control measures, regulatory compliance, and stability of exosome properties. Risks such as pathogen contamination, immune rejection, dosing variability, and inconsistent reproducibility in animal models

However, their therapeutic promise, MSCs face significant challenges in clinical translation, particularly regarding production, manufacturing, quality control, and safety. Tackling these issues demands comprehensive research and the development of systematic solutions (Fig. 7). The heterogeneous origins of MSCs lead to substantial variations in their biological characteristics and therapeutic efficacy, introducing uncertainties in clinical outcomes. Furthermore, the isolation and purification of MSCs necessitate sophisticated methodologies, and their culture conditions, including hypoxic environments and media composition, profoundly influence their proliferation potential, secretome, and immunomodulatory properties. While strategies like genetic editing and secretome optimization hold potential for improving the therapeutic efficacy of MSCs, the technical complexities and stringent regulatory requirements present significant hurdles for their development and clinical implementation. The large-scale expansion of MSCs is critical for their clinical applications, but achieving consistent quality and functionality across different batches poses significant challenges. Extended culture durations and repeated passaging can lead to changes in cell phenotype and activity, ultimately compromising therapeutic efficacy. Furthermore, cryopreservation and transportation are pivotal for preserving MSC viability and functionality. However, the cytotoxicity of traditional cryoprotectants and temperature fluctuations during storage can negatively impact their therapeutic properties (Oja et al. 2019). Clinical translation of MSCs requires adherence to stringent production standards, particularly in quality control and manufacturing standardization. Compliance with good manufacturing practice (GMP) regulations entails strict requirements for equipment, personnel, and processes, substantially elevating costs and creating additional barriers to clinical application (Lechanteur et al. 2021).

At present, the lack of a unified international standard for MSC quality assessment remains a critical issue. Variations in phenotypic markers, secretome profiling, and functional testing methodologies across institutions have led to inconsistencies in evaluating the quality and efficacy of MSC-based products (Schuessler-Lenz et al. 2023). In addition, discrepancies in regulatory approval criteria for MSC products among different countries and regions, including the FDA and EMA, have further complicated their international clinical translation.

The production and application of MSCs carry inherent risks of pathogen contamination, which directly threaten patient safety. Although preclinical studies in animal models have demonstrated the safety and efficacy of MSCs, these findings often fail to fully translate into consistent outcomes in human clinical trials, especially given the lack of standardized dosing protocols and treatment regimens. Despite their immunomodulatory properties, MSCs remain at risk of eliciting immune rejection in allogeneic transplantation, especially under conditions of prolonged treatment or high-dose administration, where the likelihood of rejection is significantly elevated. Although MSCs have shown promising safety and efficacy in preclinical animal models, these findings often fail to translate seamlessly into human clinical trials, especially given the lack of standardized dosing regimens and treatment schedules. Despite their immunomodulatory capabilities, MSCs may elicit immune rejection in allogeneic transplantation, particularly under conditions of prolonged treatment or high-dose administration, where the risk is significantly heightened.

Overcoming these barriers in MSC clinical translation necessitates coordinated efforts among scientists, industry stakeholders, and regulatory bodies. Advancements in production optimization, the establishment of global quality standards, and the strengthening of safety assessments and risk management are critical for enabling the broad clinical adoption of MSCs. These collective efforts are indispensable for unlocking the full therapeutic potential of MSCs, ultimately delivering safe, reliable, and effective treatment solutions to patients.

## Prospect

Animal studies have shown that MSC-based therapies for retinal degenerative diseases stimulate retinal cell regeneration and repair via the transplantation of MSCs and their secretome, leading to marked improvements in visual function. Early clinical trials have corroborated these findings, highlighting the potential of MSC therapies to enhance vision and improve patients’ quality of life.

Advances in materials science have created optimized microenvironments for MSCs, with both natural and cutting-edge synthetic materials serving as scaffold systems that enhance post-delivery survival and maximize therapeutic efficacy. Hydrogels, as a novel material, offer an ideal three-dimensional matrix for both in vitro culture and in vivo applications, preserving MSC pluripotency and viability during delivery, while significantly improving their long-term retention and functionality within tissues (Wechsler et al. 2021). Furthermore, by fine-tuning the physical and chemical properties of hydrogels, MSCs can demonstrate heightened adaptability, enabling them to address the therapeutic requirements of a broad range of diseases.

The field of gene editing technology is rapidly evolving, with broad applications across numerous domains. The integration of MSC therapy with gene editing technologies offers a promising strategy, enhancing MSCs’ differentiation, proliferation, and immune modulation capabilities to significantly improve therapeutic efficacy. Moreover, it enables precise manipulation of gene expression, facilitating targeted interventions for specific disease pathologies (Kimbrel and Lanza 2020). The use of CRISPR/Cas9 for genetic modification of MSCs can further enhance their ability to differentiate into desired cell types, boost their homing efficiency for accurate targeting of damaged tissues, and selectively modify specific genomic loci to augment their therapeutic potential, thereby facilitating more efficient tissue repair (Golchin et al. 2020).

MSC-Exos, with their superior biocompatibility, low immunogenicity, and capacity to traverse biological barriers, have emerged as promising nanocarriers. They enable the targeted delivery of therapeutic genes, small-molecule drugs, and macromolecular biologics to diseased sites, offering significant potential for the treatment of retinal degenerative diseases and other complex disorders. Innovations in imaging technologies have provided powerful tools for MSC-based therapies. For instance, OCT facilitates real-time tracking of the dynamic behavior of MSCs and their extracellular vesicles within tissues, including their migration, distribution, and functional activity, offering high-resolution insights for therapeutic evaluation.

Despite their promise, these innovative technologies face major challenges in practical implementation. MSC-based therapies have the potential to become a key component of retinal degenerative disease treatment by combining technological innovations with clinical practice. To achieve this, future research should focus on enhancing the functional properties of MSCs, exploring synergistic mechanisms with other therapies, and standardizing therapeutic protocols and clinical pathways. Addressing these challenges will require collaboration between scientists, industry leaders, and regulatory agencies. Realizing the full therapeutic potential of MSCs demands multifaceted innovation and collaboration across scientific, industrial, and regulatory domains. Through these collective efforts, MSC-based therapies are poised to bring transformative hope to patients with retinal diseases. As societal interest in MSC therapies continues to grow, these approaches are anticipated to evolve and offer expanded options and hope for individuals suffering from retinal degenerative diseases.

## Data Availability

No datasets were generated or analysed during the current study.
